# Pembrolizumab-based first-line treatment for PD-L1-positive, recurrent or metastatic head and neck squamous cell carcinoma: a retrospective analysis

**DOI:** 10.1186/s12885-024-12155-3

**Published:** 2024-04-08

**Authors:** Alessio Cirillo, Daniele Marinelli, Umberto Romeo, Daniela Messineo, Francesca De Felice, Marco De Vincentiis, Valentino Valentini, Silvia Mezi, Filippo Valentini, Luca Vivona, Antonella Chiavassa, Bruna Cerbelli, Daniele Santini, Paolo Bossi, Antonella Polimeni, Paolo Marchetti, Andrea Botticelli

**Affiliations:** 1 Department of Radiological, Oncological and Pathological Sciences, Sapienza University, 00161 Rome, Italy; 2 Department of Experimental Medicine, Sapienza University, 00161 Rome, Italy; 3 Department of Oral Sciences and Maxillofacial Surgery, Sapienza University, 00161 Rome, Italy; 4 Department of Sense Organs, Sapienza University, 00161 Rome, Italy; 5 Department of Medical-Surgical Sciences and Biotechnologies, Sapienza University, 04100 Latina, Italy; 6 Department of Medical and Surgical Specialties, Radiological Sciences and Public Health, University of Brescia, 25121 Brescia, Italy; 7 Istituto Dermopatico dell’Immacolata (IDI-IRCCS), 00167 Rome, Italy

**Keywords:** HNSCC, Immunotherapy, PD-L1, Biomarkers, Pembrolizumab

## Abstract

**Background:**

The KEYNOTE-048 trial showed that pembrolizumab-based first-line treatment for R/M HNSCC led to improved OS in the PD-L1 CPS ≥ 1 population when compared to the EXTREME regimen. However, the R/M HNSCC real-world population is generally frailer, often presenting with multiple comorbidities, worse performance status and older age than the population included in phase III clinical trials.

**Methods:**

This is a retrospective, single-centre analysis of patients with R/M HNSCC treated with pembrolizumab-based first-line treatment.

**Results:**

From February 2021 to March 2023, 92 patients were treated with pembrolizumab-based first-line treatment. Patients treated with pembrolizumab-based chemoimmunotherapy had better ECOG PS and younger age than those treated with pembrolizumab monotherapy. Median PFS and OS were 4 months and 8 months, respectively. PFS was similar among patients treated with pembrolizumab-based chemoimmunotherapy and pembrolizumab monotherapy, while patients treated with pembrolizumab monotherapy had worse OS (log-rank *p* =.001, HR 2.7). PFS and OS were improved in patients with PD-L1 CPS > = 20 (PFS: log-rank *p* =.005, HR 0.50; OS: log-rank *p* =.04, HR 0.57). Patients with higher ECOG PS scores had worse PFS and OS (PFS, log-rank *p* =.004; OS, log-rank *p* = 6e-04). In multivariable analysis, ECOG PS2 was associated with worse PFS and OS.

**Conclusions:**

PFS in our real-world cohort was similar to the KEYNOTE-048 reference while OS was numerically inferior. A deeper understanding of clinical variables that might affect survival outcomes of patients with R/M HNSCC beyond ECOG PS and PD-L1 CPS is urgently needed.

**Supplementary Information:**

The online version contains supplementary material available at 10.1186/s12885-024-12155-3.

## Introduction

Head and neck squamous cell carcinoma (HNSCC) encompasses a broad range of tumours arising from the oral cavity, oropharynx, hypopharynx, and larynx generally associated with tobacco use and alcohol consumption; human papillomavirus (HPV) infection was identified as a causative agent in a subgroup of HNSCC and was associated with more favourable prognosis, leading to the incorporation of the HPV-positive and HPV-negative classification into the tumour-node-metastasis (TNM) classification for HNSCC [1]. However, diagnosis of HNSCC at an early stage is uncommon, as most tumours present with locally advanced stages leading to a high risk of local recurrences and metastatic dissemination despite multimodal curative treatment. Moreover, HNSCC often presents with a significant symptom burden such as weight loss due to oral and swallowing dysfunction and speech impairment both due to local tumour spread and surgical treatment modalities [2]. 

In the phase III randomised clinical trial KEYNOTE-048 pembrolizumab-based first-line treatment for recurrent or metastatic (R/M) HNSCC led to meaningful improvements in overall survival (OS) the programmed-death ligand-1 (PD-L1) combined positive score (CPS) ≥ 1 population, when compared to cetuximab, platinum and 5-fluorouracil combination therapy (EXTREME regimen) [3]. In more detail, pembrolizumab monotherapy was shown to prolong OS when compared to the EXTREME regimen in the CPS > = 20 and > = 1 subgroups; the pembrolizumab, platinum and 5-fluorouracil regimen was shown to prolong OS in the CPS > = 20, CPS > = 1 subgroups and in the total population when compared to the EXTREME regimen. The initial OS results of KEYNOTE-048 were recently confirmed after longer follow-up, even if neither in the initial nor in the updated report pembrolizumab-based regimens showed a statistically significant improvement in progression-free survival (PFS) or overall response rate (ORR) [4]. However, as in other clinical trial of combination or single-agent immunotherapy, radiological responses in the pembrolizumab-based arms were significantly longer and OS curves reached a plateau in about 20–30% of patients at the 4-year landmark, thus highlighting that durable, long lasting responses may lead to unprecedented OS benefit. Due to the results of the KEYNOTE-048 trial, pembrolizumab-based first-line regimens in PD-L1 positive R/M HNSCC are recommended by clinical practice guidelines [5]. 

The real-world population of patients with diagnosis of R/M HNSCC is generally frailer, often presenting with multiple comorbidities, worse performance status and older age than the population included in phase III clinical trials [6–8]. It is therefore pivotal to evaluate the efficacy of pembrolizumab-based first-line treatment for R/M HNSCC in a broader population than that enrolled in phase III clinical trials: this paper shows results from a retrospective, real-world cohort study of patients with diagnosis of R/M HNSCC followed and treated at a referral centre in Italy.

## Methods

This is a retrospective, single-centre analysis of patients with diagnosis of R/M HNSCC eligible for pembrolizumab-based first-line treatment.

### Patients

All eligible patients were treated within the Medical Oncology Division at Policlinico Umberto I in Rome, Italy. All patients were treated with standard of care pembrolizumab-based first-line treatment for PD-L1 positive R/M HNSCC; eligible patients were treated either with pembrolizumab monotherapy or with pembrolizumab in combination with 5-fluorouracil and the platinum drug of choice among cisplatin and carboplatin (pembrolizumab-based chemoimmunotherapy). For patients treated with pembrolizumab-based chemoimmunotherapy, the physicians’ choice between cisplatin and carboplatin was based on patients’ renal function, comorbidites and performance status. The choice between pembrolizumab monotherapy and pembrolizumab-based chemoimmunotherapy was at the discretion of the clinician, as *per* drug label [9]. Patients with a baseline Eastern Cooperative Oncology Group performance status (ECOG PS) ranging from 0 to 2 were included in this cohort; however, a sensitivity analysis was planned on patients with a baseline ECOG PS ranging from 0 to 1 to compare results from this real-world cohort to the phase III KEYNOTE-048 trial, where only patients with baseline ECOG PS ranging from 0 to 1 were included.

Clinical data for all patients was retrieved from electronic medical records. PD-L1 CPS score analysis was performed according to clinical guidelines with the VENTANA PD-L1 (SP263) assay by a local pathologist at the Pathology Division at Policlinico Umberto I in Rome, Italy. Immune-related toxicity was defined as any immune-related adverse event (irAE) of grade > = 1 *per* Common terminology criteria for adverse events (CTCAE) version 5.0. Tumour burden was defined as any involved site including primary tumour (if not previously resected) upon clinical or radiological evaluation; tumours with up to 2 sites (including primary tumour) and 3 or more sites (including primary tumour) were grouped in separate categories.

### Statistical analysis

PFS and OS were co-primary endpoints. PFS was defined as the time from start of first-line treatment to evidence of radiological progression *per* Response Evaluation Criteria in Solid Tumours (RECIST) criteria version 1.1 or death, whichever occurred first; patients without a PFS event were censored at the last time they were known to be alive and progression-free. Overall survival was defined as the time from start of first-line treatment to death; patients without an OS event were censored at the last time they were known to be alive. Time to progression (TTP) was an exploratory endpoint and was defined as the time from start of first-line treatment to evidence of radiological progression *per* RECIST criteria; patients without a PFS event were censored at the last time they were known to be alive and progression-free; patients with a death event without previous evidence of radiological progression were censored at the time of death.

Distribution of categorical variables was tested with the Fisher’s exact test or the χ² test. Median follow-up was estimated with the reverse Kaplan-Meier method. Median survival time was estimated with the Kaplan-Meier method. Differences among groups in time-to-event endpoints were tested with unstratified log-rank tests. Hazard ratios (HR) with their relative 95% confidence intervals (95% CI) were estimated with univariable and multivariable Cox regression analyses. Variables included in multivariable Cox regression analysis were known prognostic factors selected *a priori* (ECOG PS, age) and treatment type (pembrolizumab monotherapy *versus* pembrolizumab-based chemoimmunotherapy); additionally, any tested variable with an unstratified log-rank test p-value < 0.05 was included in multivariable analysis. *Alpha* was set at 0.05 for all analyses. All analyses were performed in R version 4.3 by the corresponding author (D.M.); the following R packages were used: *dplyr, data.table, survival, survminer, prodlim, gtsummary.*

## Results

### Baseline characteristics

From February 2021 to March 2023, 92 patients were treated with pembrolizumab-based first-line treatment for CPS > = 1 R/M HNSCC. The median follow-up for the cohort was 12 months (interquartile range, 6–20 months). Thirty-five patients (38%) and 57 patients (62%) were treated with pembrolizumab-based chemoimmunotherapy and pembrolizumab monotherapy, respectively. When compared to patients treated with pembrolizumab monotherapy, patients treated with pembrolizumab-based chemoimmunotherapy were younger (*p* <.001), had lower ECOG PS scores (*p* <.001) and had less previous exposure to radiochemotherapy (*p* =.016, Table 1). There were no statistically significant differences in primary tumour location, sex, smoking status, PD-L1 CPS categories, HPV positivity and tumour burden. Among the 35 patients treated with pembrolizumab-based chemoimmunotherapy, 25 (71%) of patients received cisplatin and 10 (29%) received carboplatin.

**Table 1:** Baseline characteristics of the study population

Characteristic	Pembrolizumab plus chemotherapy, *N* = 35^1^	Pembrolizumab monotherapy, *N* = 57^1^	p-value^2^
**Primary tumor location**			**0.2**
Larynx	10 (29%)	12 (21%)	
Oral cavity	17 (49%)	36 (63%)	
Oropharynx	8 (23%)	6 (11%)	
Other/unspecified	0 (0%)	3 (5.3%)	
**Age**			**< 0.001**
< 65	21 (60%)	7 (12%)	
65–75	8 (23%)	18 (32%)	
> 75	6 (17%)	32 (56%)	
**Sex**			**> 0.9**
Female	12 (34%)	20 (35%)	
Male	23 (66%)	37 (65%)	
**ECOG PS**			**< 0.001**
0	21 (60%)	18 (32%)	
1	14 (40%)	16 (28%)	
2	0 (0%)	23 (40%)	
**PD-L1 CPS**			**> 0.9**
PD-L1 CPS 1–19	14 (40%)	23 (40%)	
PD-L1 CPS > = 20	21 (60%)	34 (60%)	
**Smoke**			**0.2**
Current smoker	20 (57%)	22 (39%)	
Never smoker	8 (23%)	18 (32%)	
Previous smoker	7 (20%)	17 (30%)	
**Previous radiochemotherapy**			**0.016**
No radiochemotherapy	32 (91%)	40 (70%)	
Previous radiochemotherapy	3 (8.6%)	17 (30%)	
**Tumor burden**			**0.2**
<=2 sites	22 (63%)	28 (49%)	
> 2 sites	13 (37%)	29 (51%)	
**HPV**			**0.2**
Negative/unknown	31 (89%)	55 (96%)	
Positive	4 (11%)	2 (3.5%)	
**Platinum agent**			**> 0.9**
Carboplatin	10 (29%)	0 (NA%)	
Cisplatin	25 (71%)	0 (NA%)	
Unknown	0	57	

^1^n (%)

^2^Fisher’s exact test; Pearson’s Chi-squared test

### Survival in patients treated with pembrolizumab-based first line treatment

**Fig. 1:**
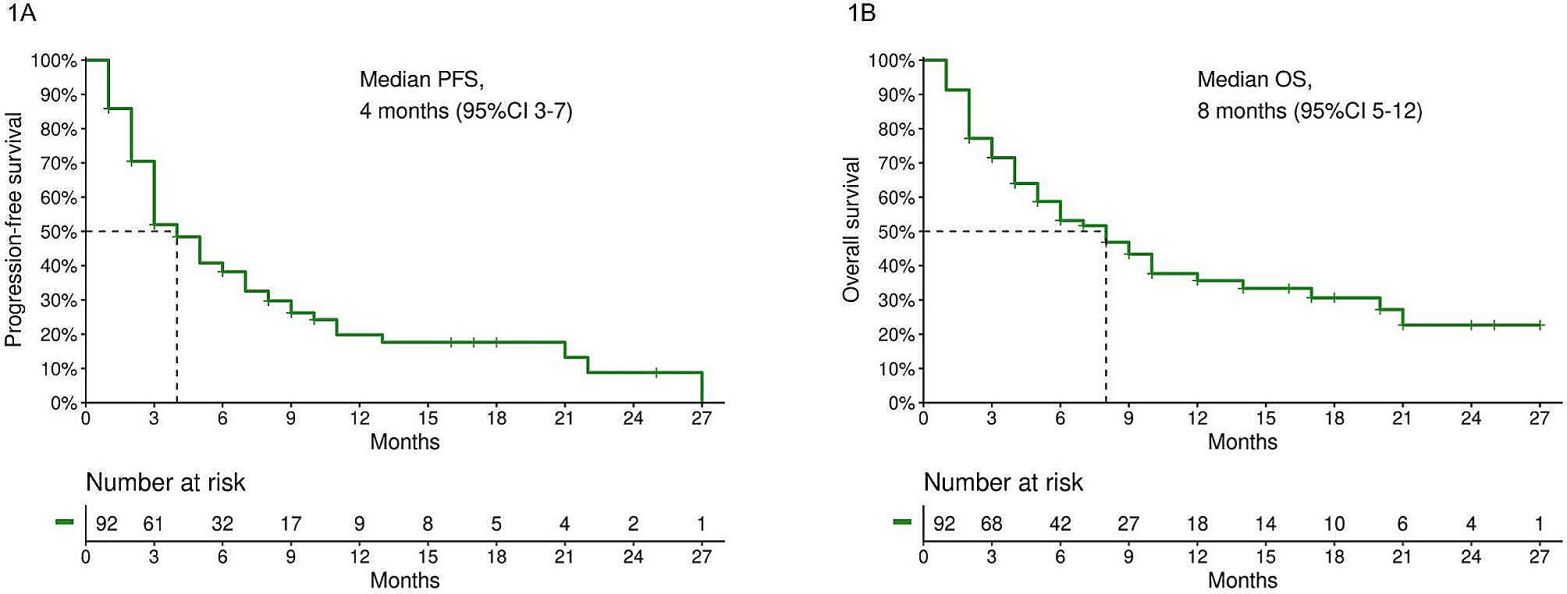
**1A**, PFS in the whole study population; **1B**, OS in the whole study population

Median progression-free survival (mPFS) and median overall survival (mOS) in the study population were 4 months (95% CI 3–7, Fig. 1A) and 8 months (95% CI 5–12, Fig. 1B), respectively; PFS at the 6 and 12 months landmarks was 38.2% (95% CI 29.2–50.1) and 19.8% (95% CI 12.1–32.6), respectively; OS at the 6 and 12 months landmarks was 53.2% (95% CI 43.4–65.1) and 35.6% (95% CI 25.7–49.3), respectively. Among patients with ECOG PS 0–1, median PFS and OS were 5 months (95% CI 4–9, Fig. S1A) and 10 months (95% CI 6-NA, Fig. S1B), respectively.

**Fig. 2:**
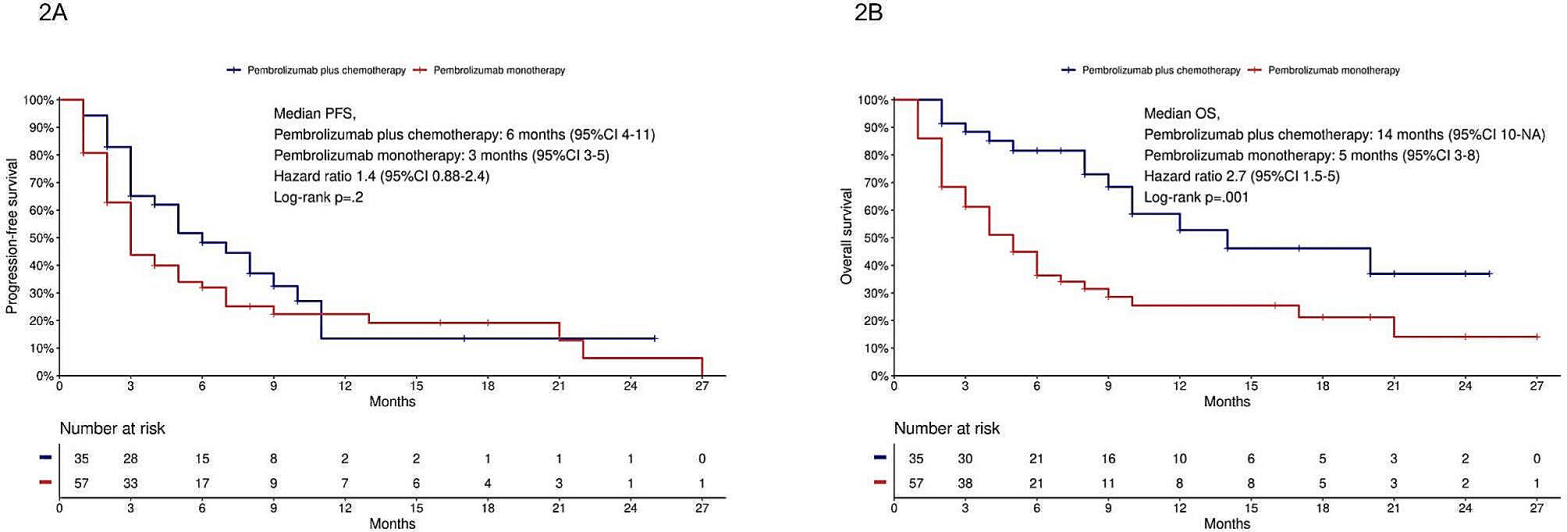
**2A**, PFS in patients treated with pembrolizumab plus chemotherapy and pembrolizumab monotherapy; **2B**, OS in patients treated with pembrolizumab plus chemotherapy and pembrolizumab monotherapy

PFS was similar among patients treated with pembrolizumab-based chemoimmunotherapy and pembrolizumab monotherapy (log-rank *p* =.2, HR 1.4, 95% CI 0.88–2.4, Fig. 2A); mPFS was 6 months (95% CI 4–11) and 3 months (95% CI 3–5), respectively. Patients treated with pembrolizumab-based chemoimmunotherapy had improved OS (log-rank *p* =.001, HR 2.7, 95% CI 1.5-5, Fig. 2B); mOS was 14 months (95% CI 10-NA) and 5 months (95% CI 3–8), respectively.

**Fig. 3:**
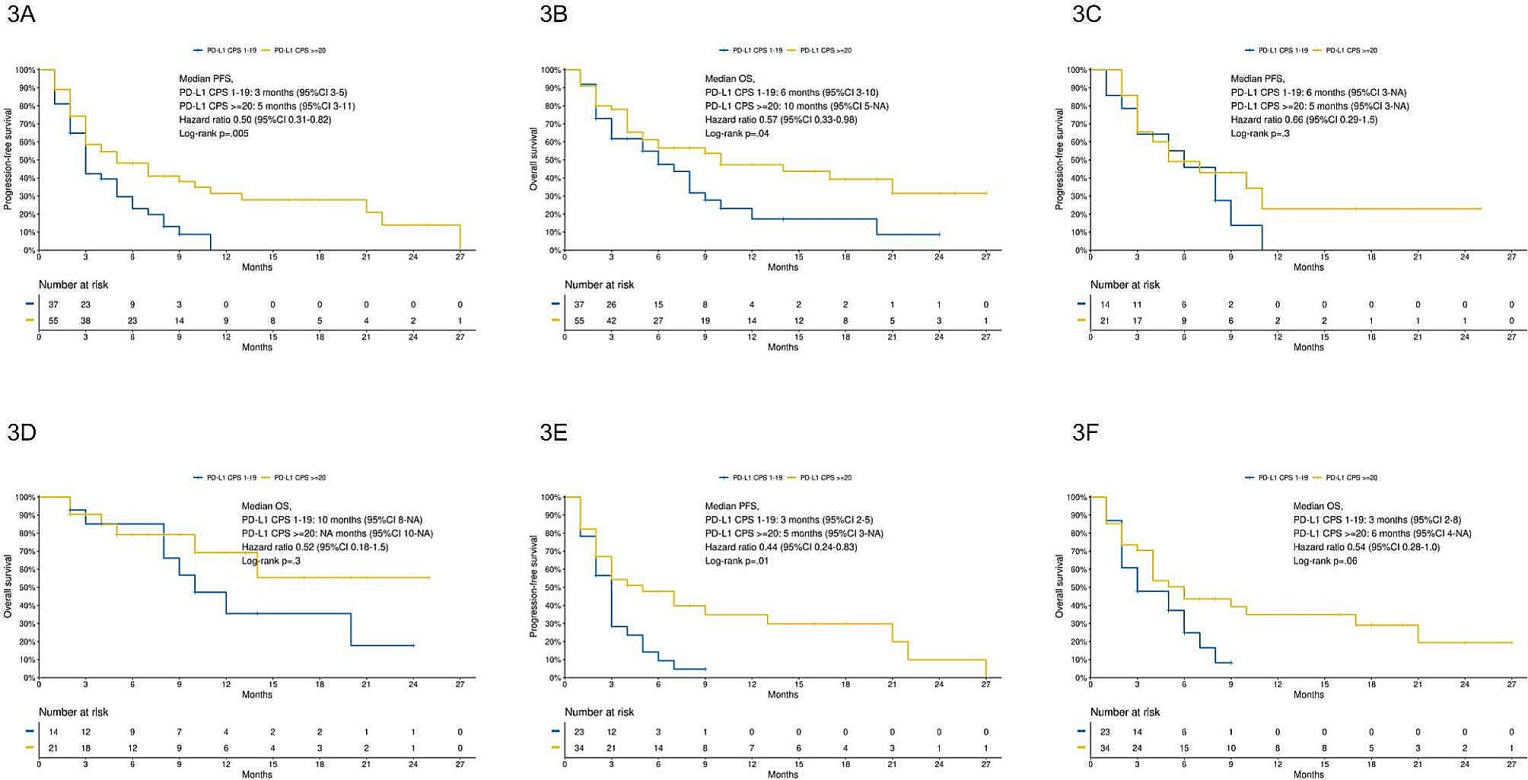
**3A**, PFS according to PD-L1 CPS classes; **3B**, OS according to PD-L1 CPS classes; **3C**, PFS in patients treated with pembrolizumab-based chemoimmunotherapy according to PD-L1 CPS classes; **3D**, OS in patients treated with pembrolizumab-based chemoimmunotherapy according to PD-L1 CPS classes; **3E**, PFS in patients treated with pembrolizumab monotherapy according to PD-L1 CPS classes; **3F**, OS in patients treated with pembrolizumab monotherapy according to PD-L1 CPS classes

PFS was improved in patients with PD-L1 CPS > = 20 when compared to patients with PD-L1 CPS 1–19 (log-rank *p* =.005, HR 0.50, 95% CI 0.31–0.82, Fig. 3A). OS was also improved in patients with PD-L1 CPS > = 20 (log-rank *p* =.04, HR 0.57, 95% CI 0.33–0.98, Fig. 3B).

In patients treated with pembrolizumab-based chemoimmunotherapy, PFS and OS were similar among patients with PD-L1 CPS 1–19 and PD-L1 CPS > = 20 (PFS: log-rank *p* =.3, HR 0.66, 95% CI 0.29–1.5, Fig. 3C; OS: log-rank *p* =.3, HR 0.52, 95% CI 0.18–1.5, Fig. 3D). However, in patients treated with pembrolizumab monotherapy, PFS was improved in patients with PD-L1 CPS > = 20 when compared to patients with PD-L1 1–19 (log-rank *p* =.01, HR 0.44, 95% CI 0.24–0.83, Fig. 3E); OS was numerically improved in patients with PD-L1 CPS > = 20 (log-rank *p* =.06, HR 0.54, 95% CI 0.28-1.0, Fig. 3F).

**Fig. 4:**
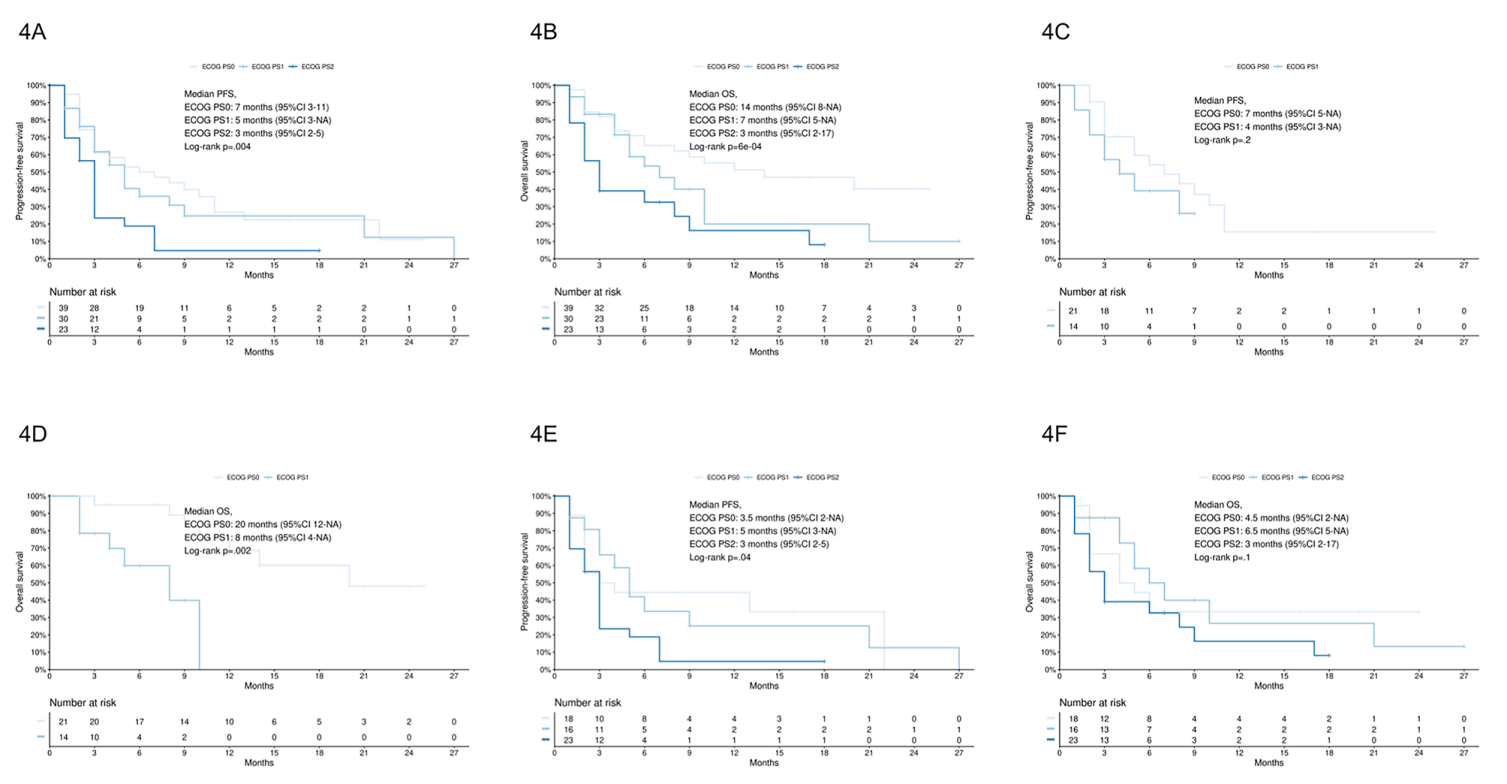
**4A**, PFS according to ECOG PS classes; **4B**, OS according to ECOG PS classes; **4C**, PFS in patients treated with pembrolizumab-based chemoimmunotherapy according to ECOG PS classes; **4D**, OS in patients treated with pembrolizumab-based chemoimmunotherapy according to ECOG PS classes; **4E**, PFS in patients treated with pembrolizumab monotherapy according to ECOG PS classes; **4F**, OS in patients treated with pembrolizumab monotherapy according to ECOG PS classes

PFS and OS were both different according to ECOG PS classes in the study population (PFS, log-rank *p* =.004, Fig. 4A; OS, log-rank *p* = 6e-04, Fig. 4B). Median PFS for patients with ECOG PS 0, 1 and 2 was 7 months (95% CI 3–11), 5 months (95% CI 3-not estimable) and 3 months (95% CI 3–5), respectively; mOS for patients with ECOG PS 0, 1 and 2 was 14 months (95% CI 8-not estimable), 7 months (95% CI 5-not estimable) and 3 months (95% CI 2–17), respectively. Among patients treated with pembrolizumab-based chemoimmunotherapy, according to ECOG PS classes, PFS was similar but OS was different (PFS, log-rank *p* =.2, Fig. 4C; OS, log-rank *p* =.002, Fig. 4D); mOS for patients with ECOG PS 0 and 1 was 20 months (95% CI 12-not estimable) and 8 months (95% CI 4-not estimable), respectively. Among patients treated with pembrolizumab monotherapy, PFS was different according to ECOG PS classes (log-rank *p* =.04, Fig. 4E) but OS was similar (log-rank *p* =.1, Fig. 4F); mPFS for patients with ECOG PS 0, 1 and 2 was 3.5 months (95% CI 2-not estimable), 5 months (95% CI 3-not estimable) and 3 months (95% CI 2–5), respectively.

PFS and OS were similar between patients with or without previous exposure to chemoradiotherapy for HNSCC (PFS, log-rank *p* =.6, Fig. S2A; OS, log-rank *p* =.6, Fig. S2B). Tumour burden classes were also not associated with differences in PFS and OS (PFS, log-rank *p* =.9, Fig. S3A; OS, log-rank *p* =.6, Fig. S3B).

**Fig. 5:**
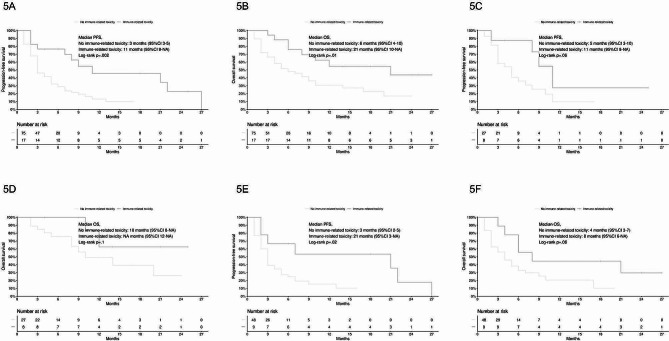
**5A**, PFS according to immune-related toxicity; **5B**, OS according to immune-related toxicity; **5C**, PFS in patients treated with pembrolizumab-based chemoimmunotherapy according to immune-related toxicity; **5D**, OS in patients treated with pembrolizumab-based chemoimmunotherapy according to immune-related toxicity; **5E**, PFS in patients treated with pembrolizumab monotherapy according to immune-related toxicity; **5F**, OS in patients treated with pembrolizumab monotherapy according to immune-related toxicity

Both PFS and OS were improved in patients with any grade irAEs (PFS, log-rank *p* =.002, Fig. 5A; OS, log-rank *p* =.01, Fig. 5B). Median PFS for patients with any grade irAEs was 11 months (95% CI 8-not estimable), while mPFS for patients without irAEs was 3 months (95% CI 3–5); mOS was 21 months (95% CI 10-not estimable) and 6 months (95% CI 4–10), respectively.

Among patients treated with pembrolizumab-based chemoimmunotherapy, PFS and OS were numerically improved in patients with any grade irAEs (PFS: log-rank *p* =.06, Fig. 5C; OS: log-rank *p* =.1, Fig. 5D). Among patients treated with pembrolizumab monotherapy, PFS was improved in patients with any grade irAEs (log-rank *p* =.02, Fig. 5E); OS was numerically improved (log-rank *p* =.06, Fig. 5F).

**Fig. 6:**
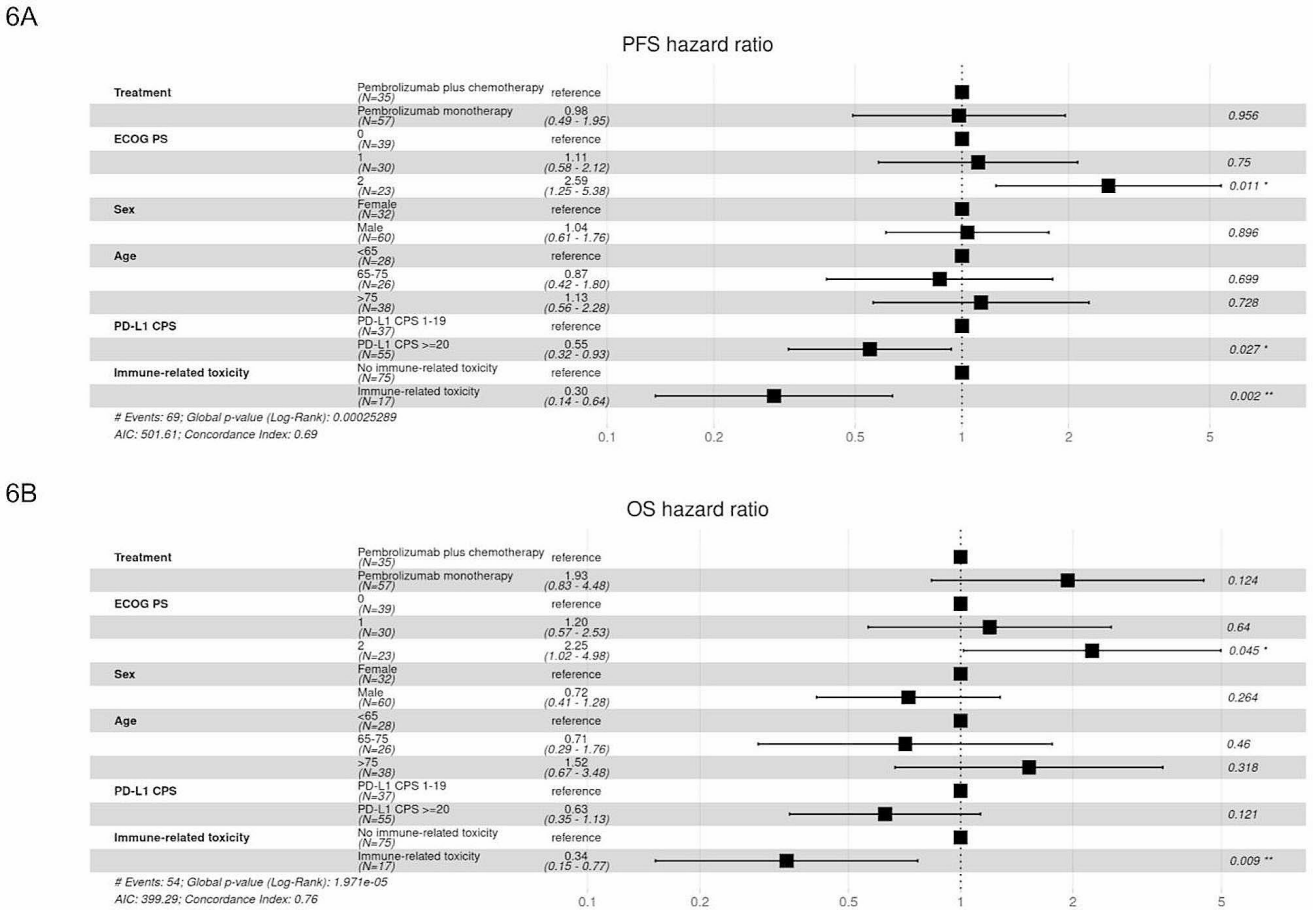
**6A**, multivariable analysis for PFS; **6B**, multivariable analysis for OS.

Multivariable Cox regression analysis for PFS showed that ECOG PS2 was associated with worse PFS while PD-L1 CPS > = 20 and any grade irAEs were associated with improved PFS (Fig. 6A). Furthermore, multivariable Cox regression analysis for OS confirmed that ECOG PS2 was associated with worse OS while any grade irAEs were associated with improved OS (Fig. 6B).

## Discussion

We showed results from a retrospective, single centre study of patients with R/M HNSCC treated with pembrolizumab-based first-line treatment at a referral centre in Italy.

Median follow-up in our cohort was 12 months, which is comparable to the median follow-up reported in the final analysis of the KEYNOTE-048 phase III trial [3]. 

Median PFS in the PD-L1 positive population of KEYNOTE-048 was 3.2 months with pembrolizumab monotherapy and 5 months with pembrolizumab-based chemoimmunotherapy; in the KEYNOTE-B10 trial, mPFS in patients treated with carboplatin, paclitaxel and pembrolizumab was 5.6 months [10]. We reported mPFS of 4 months in the whole cohort, ranging from 6 months in pembrolizumab-based chemoimmunotherapy to 3 months with pembrolizumab monotherapy; moreover, 6- and 12-months PFS landmark analyses in our cohort were fairly similar to those reported in the KEYNOTE-048 trial, with about 20% of patients being progression-free after 1 year from the start of first-line treatment. However, we acknowledge that PFS may be significantly influenced by the frequency of radiological imaging; actually, a longer time between subsequent scans is expected in a real-world cohort, potentially biassing the results towards longer PFS. Accordingly, the mOS reported in our cohort (8 months) was lower than the mOS of the pembrolizumab-based chemoimmunotherapy (13.6 months) and of the pembrolizumab monotherapy (12.3 months) arms in the KEYNOTE-048 trial [4]. The exclusion of ECOG PS2 patients in our sensitivity analysis showed mOS of 10 months among patients with ECOG PS0-1; this result is actually comparable to the results of the KEYNOTE-048 trial, even if still numerically lower. Accordingly, the landmark 12-months OS in our cohort was ∼ 36%, while in the KEYNOTE-048 trial 55% of patients in the pembrolizumab-based chemoimmunotherapy and 51% of patients in the pembrolizumab monotherapy arms were still alive after 12 months in the PD-L1 positive population. Median PFS and mOS among 23 patients in a previously published real-world cohort of R/M HNSCC treated with first-line pembrolizumab-based chemoimmunotherapy were 4 and 15 months, respectively; mPFS and mOS for first-line single-agent anti-PD1 were 4 and 10 months, respectively [11]. The results herein presented highlighted poorer survival outcomes in a real-world population when compared to the population of the reference clinical trial; nonetheless, we also highlighted that the landmark PFS and OS analyses confirmed that long-lasting disease control is achievable. However, real-world cohorts with longer follow-up are needed to confirm this observation.

Results from the ECOG PS2 category of patients are to be critically interpreted. ECOG PS 2 patients are commonly excluded from phase III clinical trials, even if they are a significant proportion of eligible patients for first-line treatment. In our cohort, ECOG PS 2 patients were present only in the pembrolizumab monotherapy group; it is likely that the safety profile of chemotherapy combinations led physicians to choose pembrolizumab monotherapy in frailer patients. ECOG PS 2 patients had the lowest mPFS and mOS in our cohort and were independently associated with worse PFS and OS in multivariable analyses; accordingly, a previous report showed shorter time on pembrolizumab-based first-line treatment for R/M HNSCC for ECOG PS 2–3 patients [12]. Moreover, in our cohort we showed that PD-L1 CPS classes significantly impacted PFS in patients treated with pembrolizumab monotherapy, with the PD-L1 CPS 1–19 class showing worse PFS. Therefore, while pembrolizumab monotherapy is likely to remain a preferred choice among ECOG PS 2 patients for its favourable safety profile, discussion of additional treatment options such as weekly chemotherapy and extensive discussion of patients’ short-term prognosis is critical in this subgroup, particularly in patients within the CPS 1–19 category [8]. Survival outcomes in patients with PD-L1 > = 20 in our cohort also compare unfavourably with the recent results from the experimental arms in the CheckMate-651 and KESTREL trials that assessed the efficacy and safety of either anti-PD1 or anti-PD1 plus anti-CTLA4 combination immunotherapy in R/M HNSCC; however, similarly to the KEYNOTE-048 trial, neither of those trials enrolled patients with ECOG PS 2. (13–14)

In our cohort we highlighted a favourable association between survival outcomes and irAEs; this was confirmed regardless of treatment category and was independently associated with better survival outcomes in multivariable analysis. An association between irAEs and improved survival outcomes was previously shown across multiple cohorts and cancer types in patients treated with immunotherapy. However, as shown in other cohorts, patients with longer exposure to immunotherapy are also more likely to experience irAEs; accordingly, we are aware that these results might be affected by immortal time bias [15–18]. 

The conclusions drawn from our study are limited by its retrospective nature; also, since the included patients were all treated at a single centre in Italy, the conclusions might not be applicable to different clinical and social contexts.

In conclusion, critical interpretation of this real-world cohort suggests that pembrolizumab-based first-line PFS was numerically comparable to the KEYNOTE-048 reference while OS was numerically inferior. Inclusion of patients with ECOG PS2 in our real-world cohort is likely to have impacted results; however, since patients with ECOG PS0-1 also had inferior OS than the patients enrolled in KEYNOTE-048, a deeper understanding of clinical variables that might affect survival outcomes of patients with R/M HNSCC beyond ECOG PS and PD-L1 CPS is urgently needed.

### Electronic supplementary material

Below is the link to the electronic supplementary material.

Supplementary Material 1

## Data Availability

The data used in the preparation of this manuscript are available from the corresponding author upon reasonable request.

## References

[1] Johnson DE, Burtness B, Leemans CR, Lui VWY, Bauman JE, Grandis JR. Head and neck squamous cell carcinoma. Nat Rev Dis Primers. 2020;6(1):92. 10.1038/s41572-020-00224-3. Erratum in: Nat Rev Dis Primers. 2023;9(1):4. PMID: 33243986; PMCID: PMC7944998. 10.1038/s41572-020-00224-3 PMC7944998 33243986

[2] Chow LQM. Head and Neck Cancer. N Engl J Med. 2020;382(1):60–72. 10.1056/NEJMra1715715. PMID: 31893516. 10.1056/NEJMra1715715 31893516

[3] Burtness B, Harrington KJ, Greil R, Soulières D, Tahara M, de Castro G Jr, Psyrri A, Basté N, Neupane P, Bratland Å, Fuereder T, Hughes BGM, Mesía R, Ngamphaiboon N, Rordorf T, Wan Ishak WZ, Hong RL, González Mendoza R, Roy A, Zhang Y, Gumuscu B, Cheng JD, Jin F, Rischin D. KEYNOTE-048 Investigators. Pembrolizumab alone or with chemotherapy versus cetuximab with chemotherapy for recurrent or metastatic squamous cell carcinoma of the head and neck (KEYNOTE-048): a randomised, open-label, phase 3 study. Lancet. 2019;394(10212):1915–1928. 10.1016/S0140-6736(19)32591-7. Epub 2019 Nov 1. Erratum in: Lancet. 2020;395(10220):272. Erratum in: Lancet. 2020;395(10224):564. Erratum in: Lancet. 2021;397(10291):2252. PMID: 31679945.

[4] Harrington KJ, Burtness B, Greil R, Soulières D, Tahara M, de Castro G, Psyrri A, Brana I, Basté N, Neupane P, Bratland Å, Fuereder T, Hughes BGM, Mesia R, Ngamphaiboon N, Rordorf T, Wan Ishak WZ, Lin J, Gumuscu B, Swaby RF, Rischin D (2023). Pembrolizumab with or without chemotherapy in recurrent or metastatic Head and Neck squamous cell carcinoma: updated results of the phase III KEYNOTE-048 study. J Clin Oncol.

[5] Machiels JP, René Leemans C, Golusinski W, Grau C, Licitra L, Gregoire V (2020). EHNS Executive Board. Electronic address: secretariat@ehns.org; ESMO Guidelines Committee. Electronic address: clinicalguidelines@esmo.org; ESTRO Executive Board. Electronic address: info@estro.org. Squamous cell carcinoma of the oral cavity, larynx, oropharynx and hypopharynx: EHNS-ESMO-ESTRO Clinical Practice guidelines for diagnosis, treatment and follow-up. Ann Oncol.

[6] Corrêa GT, Bandeira GA, Cavalcanti BG, Santos FB, Rodrigues Neto JF, Guimarães AL, Haikal DS, De Paula AM. Analysis of ECOG performance status in head and neck squamous cell carcinoma patients: association with sociodemographical and clinical factors, and overall survival. Support Care Cancer. 2012;20(11):2679-85. 10.1007/s00520-012-1386-y. PMID: 22314971. 10.1007/s00520-012-1386-y 22314971

[7] Szturz P, Bossi P, Vermorken JB (2019). Systemic treatment in elderly head and neck cancer patients: recommendations for clinical practice. Curr Opin Otolaryngol Head Neck Surg.

[8] Botticelli A, Pomati G, Cirillo A, Mammone G, Ciurluini F, Cerbelli B, Sciattella P, Ralli M, Romeo U, De Felice F, Catalano C, Vullo F, Della Monaca M, Amirhassankhani S, Tomao S, Valentini V, De Vincentiis M, Tombolini V, Della Rocca C, Polimeni A, di Gioia C, Corsi A, D’Amati G, Mezi S, Marchetti P (2021). Weekly chemotherapy as first line treatment in frail head and neck cancer patients in the immunotherapy era. J Transl Med.

[9] https://www.ema.europa.eu/en/documents/product-information/keytruda-epar-product-information_en.pdf.

[10] Dzienis MR, Cundom JE, Fuentes CS, Hansen AR, Nordlinger MJ, Pastor AV, Oppelt P, Neki A, Gregg RW, Lima IPF, Franke FA, da Cunha Junior GF, Tseng JE, Loree T, Joshi AJ, Mccarthy JS, Naicker N, Sidi Y, Gumuscu B, De Castro (2022). G. 651O - pembrolizumab (pembro) + carboplatin (carbo) + paclitaxel (pacli) as first-line (1L) therapy in recurrent/metastatic (R/M) head and neck squamous cell carcinoma (HNSCC): phase VI KEYNOTE-B10 study. Ann Oncol.

[11] Wagner SM, Magnes T, Melchardt T, Kiem D, Weiss L, Neureiter D, Wagner C, Aretin MB, Nemec S, Gamerith G, Pall G, Greil R, Fuereder T. Real-world Data of Palliative First-line Checkpoint Inhibitor Therapy for Head and Neck Cancer. Anticancer Res. 2023;43(3):1273–1282. 10.21873/anticanres.16274. PMID: 36854497. 10.21873/anticanres.16274 36854497

[12] Black CM, Wang L, Ramakrishnan K, Turzhitsky V. Real-world time on treatment analysis of pembrolizumab treated recurrent/metastatic head & neck squamous cell carcinoma (R/M HNSCC) patients stratified by ECOG PS in the United States. J Clin Oncol 2022 40:16_suppl, e18007–18007.

[13] Haddad RI, Harrington K, Tahara M, Ferris RL, Gillison M, Fayette J, Daste A, Koralewski P, Zurawski B, Taberna M, Saba NF, Mak M, Kawecki A, Girotto G, Alvarez Avitia MA, Even C, Toledo JGR, Guminski A, Müller-Richter U, Kiyota N, Roberts M, Khan TA, Miller-Moslin K, Wei L, Argiris A (2023). Nivolumab Plus Ipilimumab Versus EXTREME Regimen as First-Line treatment for Recurrent/Metastatic squamous cell carcinoma of the Head and Neck: The Final results of CheckMate 651. J Clin Oncol.

[14] Psyrri A, Fayette J, Harrington K, Gillison M, Ahn MJ, Takahashi S, Weiss J, Machiels JP, Baxi S, Vasilyev A, Karpenko A, Dvorkin M, Hsieh CY, Thungappa SC, Segura PP, Vynnychenko I, Haddad R, Kasper S, Mauz PS, Baker V, He P, Evans B, Wildsmith S, Olsson RF, Yovine A, Kurland JF, Morsli N, Seiwert TY, KESTREL Investigators (2023). Durvalumab with or without tremelimumab versus the EXTREME regimen as first-line treatment for recurrent or metastatic squamous cell carcinoma of the head and neck: KESTREL, a randomized, open-label, phase III study. Ann Oncol.

[15] Foster CC, Couey MA, Kochanny SE, Khattri A, Acharya RK, Tan YC, Brisson RJ, Leidner RS, Seiwert TY (2021). Immune-related adverse events are associated with improved response, progression-free survival, and overall survival for patients with head and neck cancer receiving immune checkpoint inhibitors. Cancer.

[16] Conroy M, Naidoo J (2022). Immune-related adverse events and the balancing act of immunotherapy. Nat Commun.

[17] Yadav K, Lewis RJ (2021). Immortal Time Bias in Observational studies. JAMA.

[18] Dall’Olio FG, Di Nunno V, Massari F (2020). Immortal Time Bias question in the Association between toxicity and outcome of Immune Checkpoint inhibitors. J Clin Oncol.

