# Correction: Pembrolizumab-based first-line treatment for PD-L1-positive, recurrent or metastatic head and neck squamous cell carcinoma: a retrospective analysis

**DOI:** 10.1186/s12885-024-12249-y

**Published:** 2024-04-17

**Authors:** Alessio Cirillo, Daniele Marinelli, Umberto Romeo, Daniela Messineo, Francesca De Felice, Marco De Vincentiis, Valentino Valentini, Silvia Mezi, Filippo Valentini, Luca Vivona, Antonella Chiavassa, Bruna Cerbelli, Daniele Santini, Paolo Bossi, Antonella Polimeni, Paolo Marchetti, Andrea Botticelli

**Affiliations:** 1 Department of Radiological, Oncological and Pathological Sciences, Sapienza University, 00161 Rome, Italy; 2 Department of Experimental Medicine, Sapienza University, 00161 Rome, Italy; 3 Department of Oral Sciences and Maxillofacial Surgery, Sapienza University, 00161 Rome, Italy; 4 Department of Sense Organs, Sapienza University, 00161 Rome, Italy; 5 Department of Medical-Surgical Sciences and Biotechnologies, Sapienza University, 04100 Latina, Italy; 6 Department of Medical and Surgical Specialties, Radiological Sciences and Public Health, University of Brescia, 25121 Brescia, Italy; 7 Istituto Dermopatico Dell’Immacolata (IDI-IRCCS), 00167 Rome, Italy

**Correction** **:** **BMC Cancer 24, 430 (2024)**

**https://doi.org/10.1186/s12885-024-12155-3**

Following publication of the original article [1], the authors noticed that Fig. 4 was a duplicate of Fig. 3.

This correction article consists of the correct Fig. 3 and Fig. 4.

**Fig. 3:**
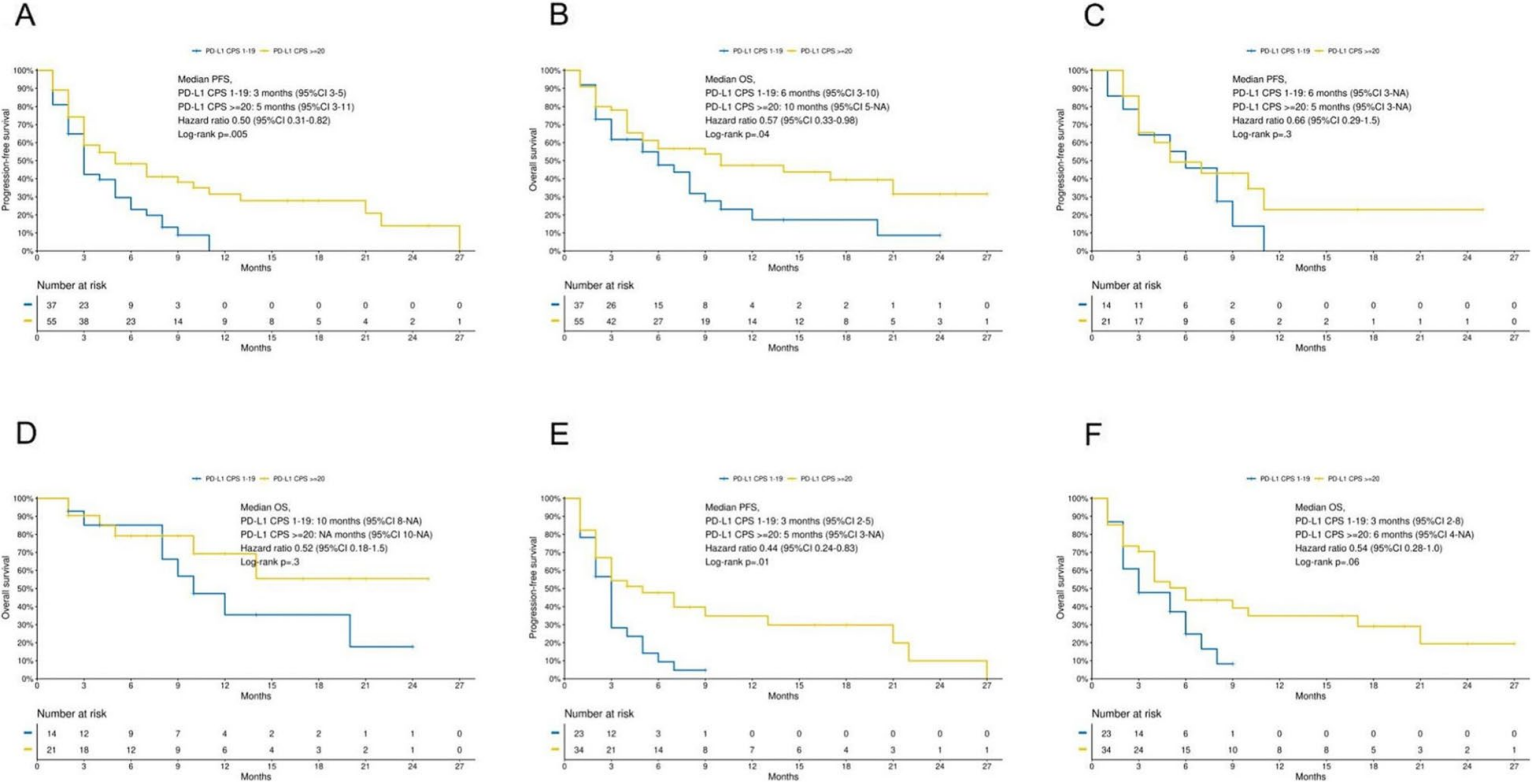
**3A**, PFS according to PD-L1 CPS classes; **3B**, OS according to PD-L1 CPS classes; **3C**, PFS in patients treated with pembrolizumab-based chemoimmunotherapy according to PD-L1 CPS classes; **3D**, OS in patients treated with pembrolizumab-based chemoimmunotherapy according to PD-L1 CPS classes; **3E**, PFS in patients treated with pembrolizumab monotherapy according to PD-L1 CPS classes; **3F**, OS in patients treated with pembrolizumab monotherapy according to PD-L1 CPS classes

**Fig. 4:**
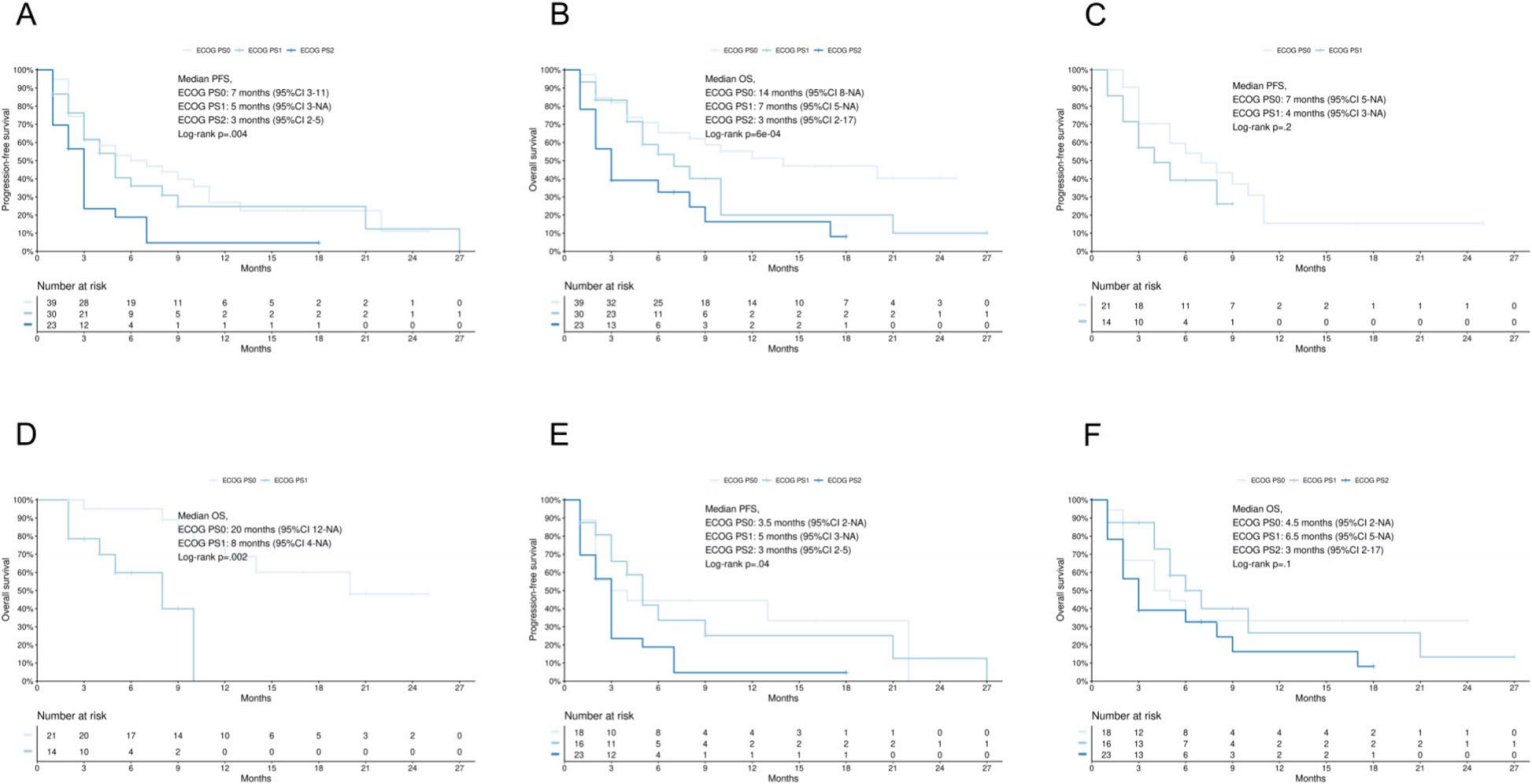
**4A**, PFS according to ECOG PS classes; **4B**, OS according to ECOG PS classes; **4C**, PFS in patients treated with pembrolizumab-based chemoimmunotherapy according to ECOG PS classes; **4D**, OS in patients treated with pembrolizumab-based chemoimmunotherapy according to ECOG PS classes; **4E**, PFS in patients treated with pembrolizumab monotherapy according to ECOG PS classes; **4F**, OS in patients treated with pembrolizumab monotherapy according to ECOG PS classes

The original article [1] has been corrected.

## References

[1] Cirillo A, Marinelli D, Romeo U (2024). Pembrolizumab-based first-line treatment for PD-L1-positive, recurrent or metastatic head and neck squamous cell carcinoma: a retrospective analysis. BMC Cancer.

