# Supplementary material for: Pembrolizumab-based first-line treatment for PD-L1-positive, recurrent or metastatic head and neck squamous cell carcinoma: a retrospective analysis
Source: BMC Cancer. 2024 Apr 8;24:430. doi: 10.1186/s12885-024-12155-3 (PMC11000280; doi:10.1186/s12885-024-12155-3)
Supplement: Supplementary file 1 — Supplementary Material 1 [file 12885_2024_12155_MOESM1_ESM.docx]

**Figure S1. S1A, PFS in patients with ECOG PS0-1; S1B, OS in the patients with ECOG PS0-1.**

**
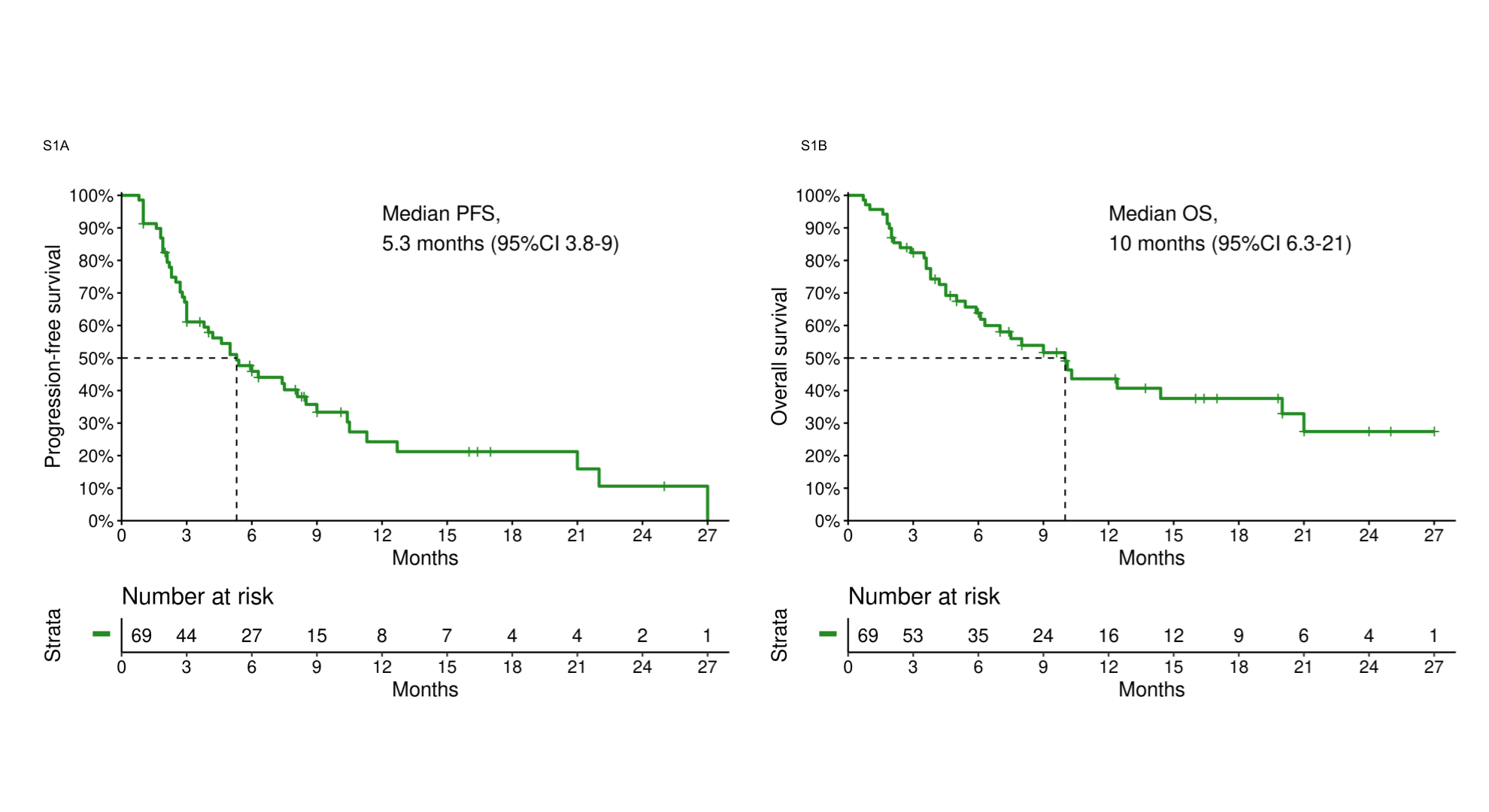
**

**Figure S2. S2A, PFS according to previous exposure to chemoradiotherapy; S2B, OS according to previous chemoradiotherapy.**

**
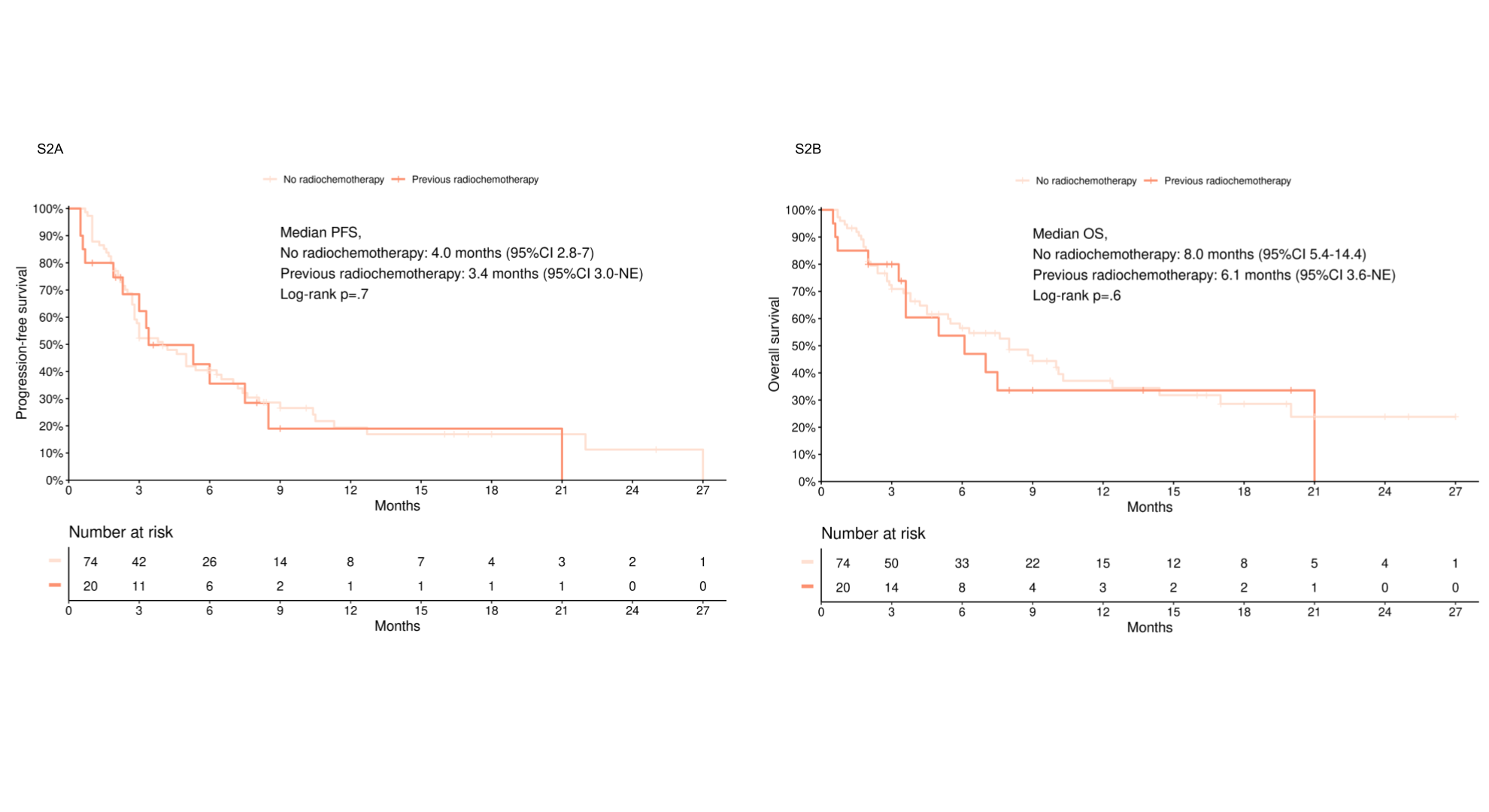
**

**Figure S3. S3A, PFS according to tumour burden; S3B, OS according to tumour burden.**

**
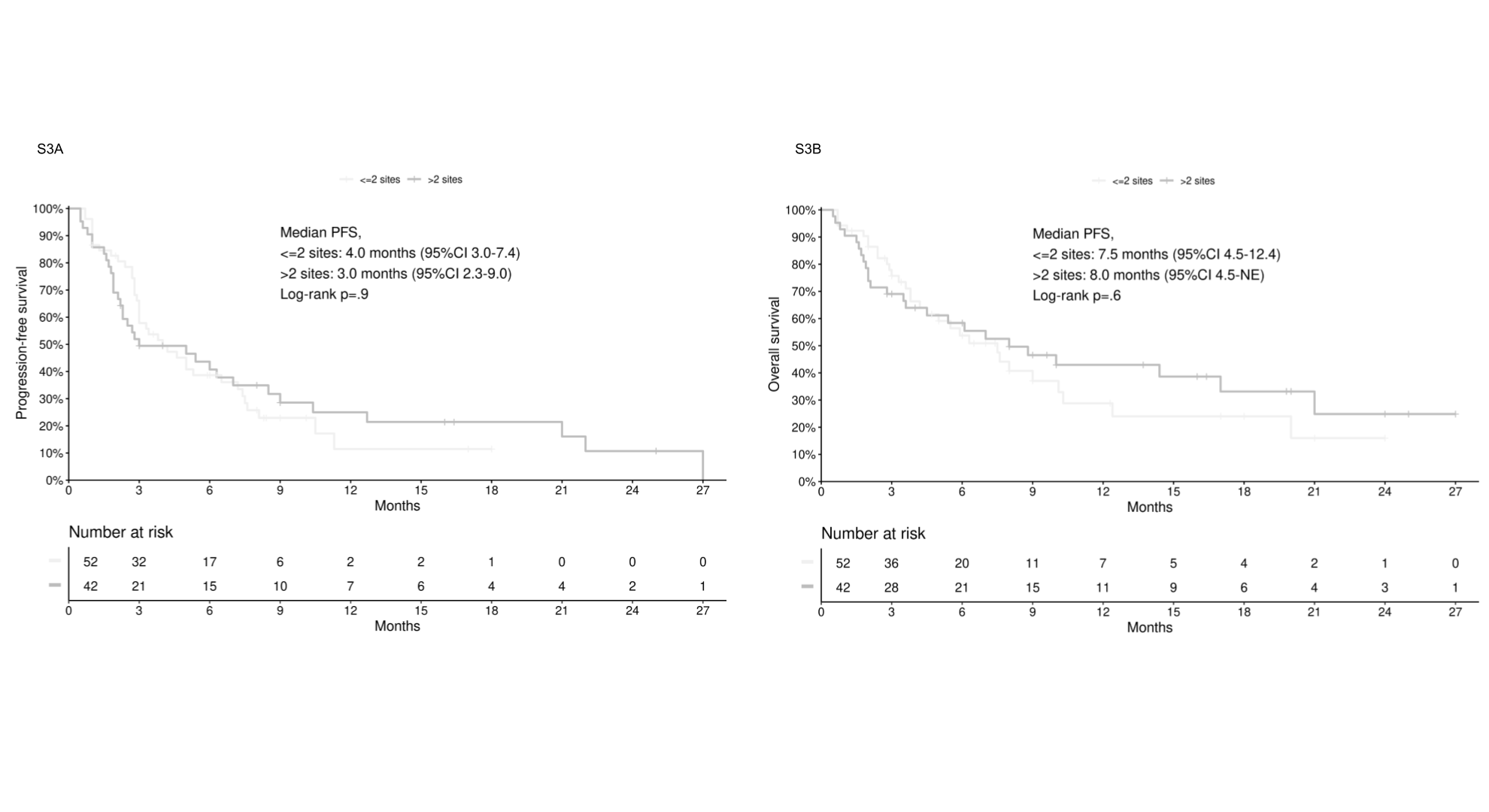
**

**Figure S4. Time-to-progression analysis according to treatment type.**

**
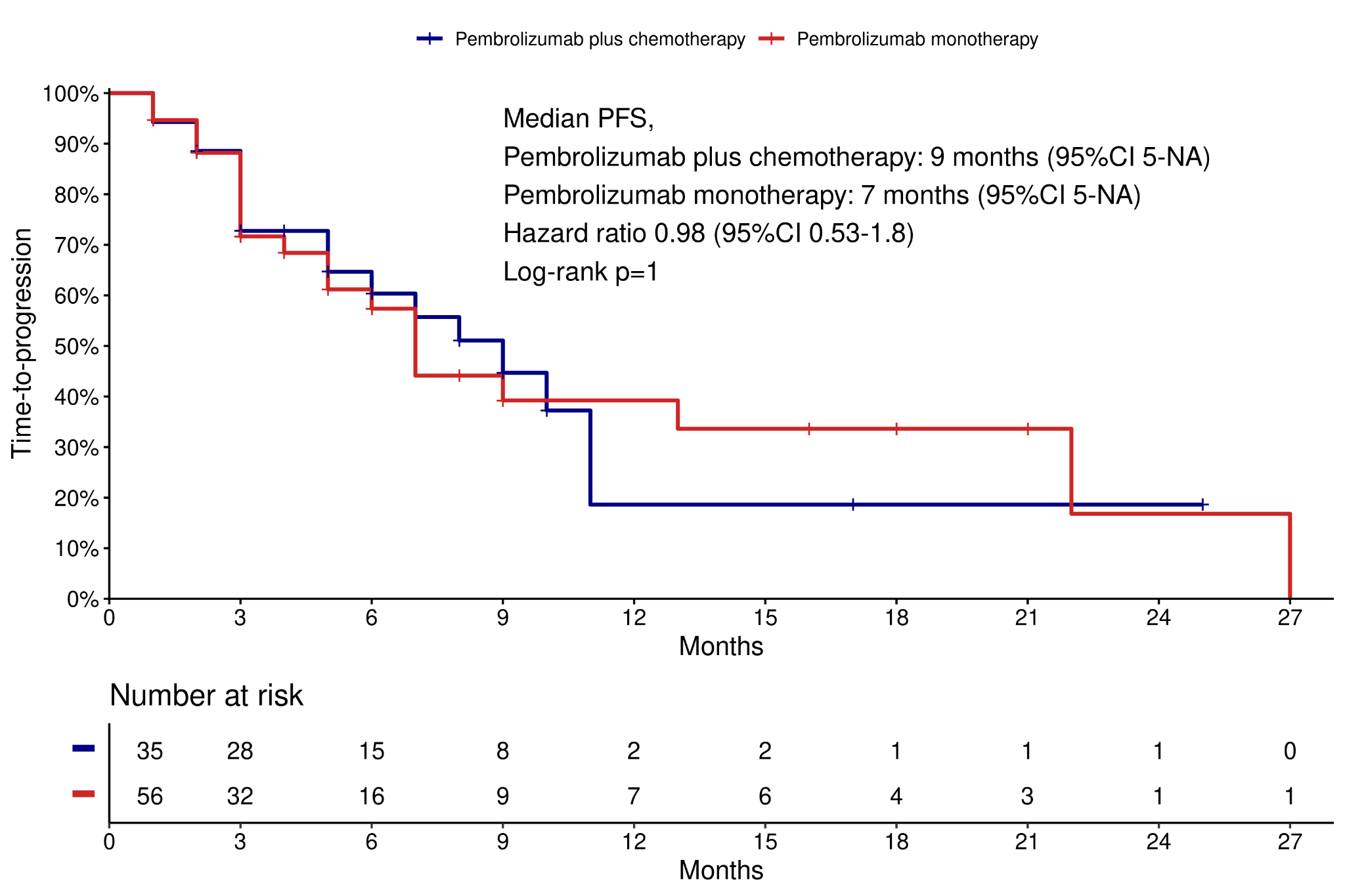
**
